# Treatment of methotrexate (MTX) intolerance: behavioural therapy, versus switch to parenteral MTX versus oral MTX

**DOI:** 10.1186/1546-0096-9-S1-O23

**Published:** 2011-09-14

**Authors:** M Verkaaik, M Bulatović, G Sinnema, C Rademaker, NM Wulffraat

**Affiliations:** 1 University Medical Center Utrecht, Wilhelmina Children’s Hospital, Department of Pediatric Immunology, Utrecht, The Netherlands

## Background

More than 50% of JIA patients on Methotrexate (MTX) suffer from MTX-related gastrointestinal adverse effects – MTX intolerance (1). Nevertheless, the best approach to treat MTX intolerance is unknown.

## Aim

To compare the effect of behavioural therapy or a switch to parenteral MTX with oral MTX on MTX intolerance.

## Methods

45 JIA patients with MTX intolerance were randomised to receive oral MTX with anti-emetics (standard of care), parenteral MTX or oral MTX with behavioural therapy. Primary outcome was the occurrence of MTX intolerance, defined as ≥5 points on a validated MISS questionnaire, after a 3-month intervention period. Secondary outcome measures were: MTX intolerance after 6 and 12 months and the number of patients that discontinued MTX or switched to another treatment arm due to intolerance.

## Results

After 3 months MTX intolerance resolved in 6 (54.5%) patients on standard of care, 4 (57.1%) patients on parenteral MTX and 4 (36.4%) patients on behavioural therapy. Nine patients (30.0%) discontinued MTX or switched to another treatment arm due to MTX intolerance. Of remaining patients at 6 months, MTX intolerance resolved in 2 (50%), 4 (80%) and 4 (57.1%) patients, and at 12 months in 2 (66.7%), 4 (80%) and 3 (50%) patients in three treatment groups respectively. All treatment arms showed a marked decrease in MTX intolerance score on the MISS questionnaire from on average 15 points to 6 points after 3 months.

## Conclusion

Behavioural therapy and parenteral MTX were not more effective than the standard of care in treating MTX intolerance. Instead, all treatments strongly diminished MTX intolerance, probably due to changes in cognitions and self-efficacy. Physicians should not discontinue MTX or switch to biologicals in case of MTX intolerance, but rather strengthen the patient’s self-efficacy to cope with MTX intolerance. We propose several strategies to this end.

## References

[B1] Bulatovic M Heijstek MW Verkaaik M van Dijkhuizen EH Armbrust W Hoppenreijs EP High prevalence of methotrexate intolerance in juvenile idiopathic arthritis: Development and validation of a methotrexate intolerance severity score Arthritis Rheum 2011 Epub ahead of print 10.1002/art.30367 21437879

